# The quality and effectiveness of Social-Emotional Learning (SEL) intervention studies in Korea: A meta-analysis

**DOI:** 10.1371/journal.pone.0269996

**Published:** 2022-06-24

**Authors:** Dongil Kim, Jin Hyung Lim, Jechun An

**Affiliations:** 1 Department of Education, Seoul National University, Seoul, Republic of Korea; 2 Department of Educational Psychology, University of Minnesota at Twin Cities, Minneapolis, Minnesota, United States of America; Central China Normal University, CHINA

## Abstract

Social-emotional learning (SEL) is an educational model for improving social-emotional competences of all students and a long-term education program connecting school, home, and community. Although there has been active research to establish evidence-based practice (EBP) of SEL programs worldwide, the quality of SEL intervention studies which is an integral part of evaluating EBP was rarely investigated. In addition, prior meta-analytic studies focused only on the effectiveness of SEL programs conducted in Western society. In this sense, in order to contribute to establishing EBP of SEL programs, the current research sought to analyze both quality and effectiveness of SEL intervention studies conducted in Korea where SEL has been investigated and applied in classroom since 2010. To conduct this study, we selected 22 peer-reviewed articles (about 23 SEL programs) and analyzed their quality by Evidence-Based Intervention (EBI) indicators and calculated effect sizes using a meta-analysis. The results of the quality analysis revealed that SEL intervention studies had some limitations with a statistical analysis, use of measurement, a control group design, intervention fidelity, and external validity. The global effect size of SEL programs was 0.27, and the results from the effect size analyses by controlling variables showed that group compositions, the number of sessions, and session length were accountable for the variability of effect sizes. Based on these findings, we discussed the directions for future research and practice on the EBP of SEL programs that can be appreciated by researchers worldwide.

## Introduction

Social-emotional learning (SEL) represents an educational model for improving social-emotional competences of all students and is known as a long-term education program connecting school, family, and community [1]. SEL aims to promote five core competencies including self-awareness, self-management, social awareness, relationship skills, and responsible decision-making [2]. By improving those competencies, children and adolescents facilitate their prosocial behaviors, positive social relationships, and academic achievement, as well as reduce conduct problems and emotional distress [3]. Research also indicates that SEL programs are likely to have positive long-term outcomes both in academic success and mental health for individuals after leaving school [4]. In addition, due to an increasing emphasis on social and emotional competences being as important as academic success during childhood and adolescence, school-based SEL is now actively implemented through a variety of intensities, formats, cultures, and countries [5].

However, individual SEL programs do not always show the same astonishing improvement in students’ socioemotional competencies unless implemented effectively, with high-quality, evidence-based instructions [5, 6]. In educational practice, since teachers are required to use instructions scientifically proven effective, it is necessary for researchers to constantly identify and implement evidence-based practice (EBP) [7, 8]. In order to investigate EBP of a certain kind of educational program, it is first necessary to identify the considerable effectiveness of that program across various participants, formats, and educational settings [9]. In this sense, a meta-analysis, statistically synthesizing the results from a multitude of studies on SEL, is required to establish EBP of SEL programs.

There have been several meta-analytic studies to examine the overall effectiveness of SEL and investigate EBP. For example, Durlak et al. [10] synthesized effectiveness of 213 school-based universal SEL programs on students’ (K-12) social-emotional skills, attitudes, behaviors, and academic performances. During the meta-analysis, they inserted dichotomously coded characteristics of intervention (e.g., whether the intervention met Sequenced, Active, Focused, and Explicit [SAFE] criteria) and implementers (e.g., whether authors monitored the process of intervention) as moderators of SEL programs’ effects on student outcomes. It was found that SEL programs fulfilling SAFE criteria and showing no problems in implementation had larger effect sizes than those not [10]. Wigelsworth et al. [5] also analyzed the effectiveness of school-based universal SEL programs on students’ social-emotional competencies, self-attitudes, prosocial behaviors, conduct problems, emotional distress, and academic achievement. However, they used different moderators to establish EBP from those used in Durlak et al. [10]: (1) stage of evaluation (efficacy or effectiveness); (2) involvement from the program developer in the evaluation (led, involved, or independent); and (3) whether the program was implemented in its original country (home or away). Although the hypotheses were promising, the moderating effects were not consistent across different learning outcomes and further research was needed.

There were two additional meta-analyses which focused on the effects of SEL on social-emotional competences in early childhood. Blewitt et al. [11] examined 79 studies on universal curriculum-based SEL programs delivered to children aged 2–6 years, and showed their small- to moderate-sized effects on social-emotional competences, behavioral self-regulation, emotional and behavioral problems, and early academic performance. Those learning outcomes were moderated by intervention leader, type of assessment, informant, child age, and study quality [11]. Murano, Sawyer, and Lipnevich [12] also summarized the effects of 48 SEL programs on social-emotional skills and problem behaviors of preschool children, but they included targeted (selective) programs as well as universal ones. On average, targeted SEL programs had larger effect sizes than universal ones, and those effects were moderated by student-level, program-level, and methodological factors [12].

Furthermore, since SEL aims to have positive long-term effects in students’ lives, Taylor et al. [4] reviewed 82 school-based universal SEL instructions delivered to K-12 students and emphasized the follow-up effects of those programs both on social-emotional skills and academic achievement. It should be noted that positive outcomes were similar across different races, socio-economic statuses, and school locations [4]. Lastly, Corcoran et al. [13] explored the effects of a total of 40 PreK-12 school-based SEL interventions on students’ reading, math, and science achievement. Although the study found that SEL provided significant positive effect on students’ academic achievement, researchers were concerned that programs using more rigorous randomized methods might not fully support meaningful effects as once thought [13].

Despite continuing efforts to establish EBP for SEL interventions, there are some limitations that are not addressed by the prior meta-analytic studies that we have reviewed. First, former literature reviews on SEL programs did not fully examine scientific quality of each implementation. In order to investigate whether the specific intervention have some scientific evidence, it is imperative to evaluate the quality of the individual studies in terms of study design and method as well as identifying their effectiveness [8, 14]. Although prior meta-analyses [5, 10–13] considered methodology and study design as possible moderators for the effect sizes, they did not examine exactly what needs to be complemented in order to improve overall quality of intervention studies and suggest scientific evidence for SEL implementers. Secondly, in spite of Wigelsworth et al. [5] investigating the impact of cultural transferability on the effectiveness of SEL programs, it was still confined to English-speaking countries and continents such as North America, Australia, and Europe. Therefore, we do not know whether SEL interventions implemented in places other than America, Australia, and Europe have the same levels of effectiveness.

In Korea, the concept of SEL was introduced in the late 2000s and theoretical discussions for the necessity of SEL have been ongoing in the field of ethics education [15, 16]. In addition, researchers in diverse subject education (e.g., Korean, math, social studies, and music education) have strived to develop curricula integrating the main contents of each subject and the core values of SEL [17–20]. Since 2010, intervention studies were also conducted to confirm the effectiveness of SEL programs delivered to K-12 students in diverse classroom settings. Reviewing those intervention studies, SEL programs in Korea were mostly implemented for elementary school students without disabilities. The programs used in those studies can be categorized into effect-proven programs by replicated studies [21, 22] and programs developed by researchers themselves [23, 24], as well as programs applied to subject education [25, 26] and those implemented with extra-curricular activities [21–24]. Numerous participants from those programs showed positive effects in the improvement of academic attitudes as well as social-emotional competences.

As diverse SEL programs have been developed and implemented in Korea for the last 10 years, the current study aims to evaluate the EBP of SEL programs in Korea from two perspectives. First, in order to complement prior meta-analytic studies, this study examined the quality of collected literature using Evidence-Based Intervention (EBI) indicators by Kratochwill & Stoiber [27]. EBI indicators were often used to review programs for children and adolescents in the field of school psychology evaluating the areas of statistical methods, measurement, group design, external validity, and intervention fidelity [28]. Furthermore, since EBI indicators are based on a scientist-practitioner model to narrow the gap between research and practice in education [29], quality analysis from these indicators would surely improve the possibility for EBP to be more actively applied in educational settings. However, it should be noted that these indicators suggest recommendations to follow when designing experimental studies, instead of an absolute standard to determine whether a certain study is unacceptable. Therefore, the aim of the quality analysis is not to reprimand intervention studies that did not fulfill EBI criteria, but to suggest some future directions for improving overall quality of SEL studies.

Secondly, a meta-analysis is also required to identify the most effective practice for SEL, explain variation of effect sizes by controlling variables as well as a global effect size. In order to optimize the applicability in real school settings, the current study used types of participants, instruction programs, and learning outcomes as controlling variables [30], and discussed how SELs need to be complemented based on the results. This meta-analytic study would benefit not only Korean educators but also SEL implementers worldwide. It is because SEL programs conducted in Korea are also based on contents and instructional strategies recommended by CASEL [2], which is identical to those implemented in North America, Australia, and Europe. By synthesizing former studies on SEL programs in Korea, it is expected to initiate discussions on worldwide SEL practices otherwise confined to Western educational settings. Therefore, the present study poses the following research questions.

RQ1: What is the quality of experimental studies on SEL implemented in Korea?
RQ2: What is the global effect size on SEL programs included in the meta-analysis?
RQ3: To what extent did participant-, program-related variables, and types of learning outcomes moderate the effectiveness of SEL?

## Methods

### Literature search

**Fig 1:**
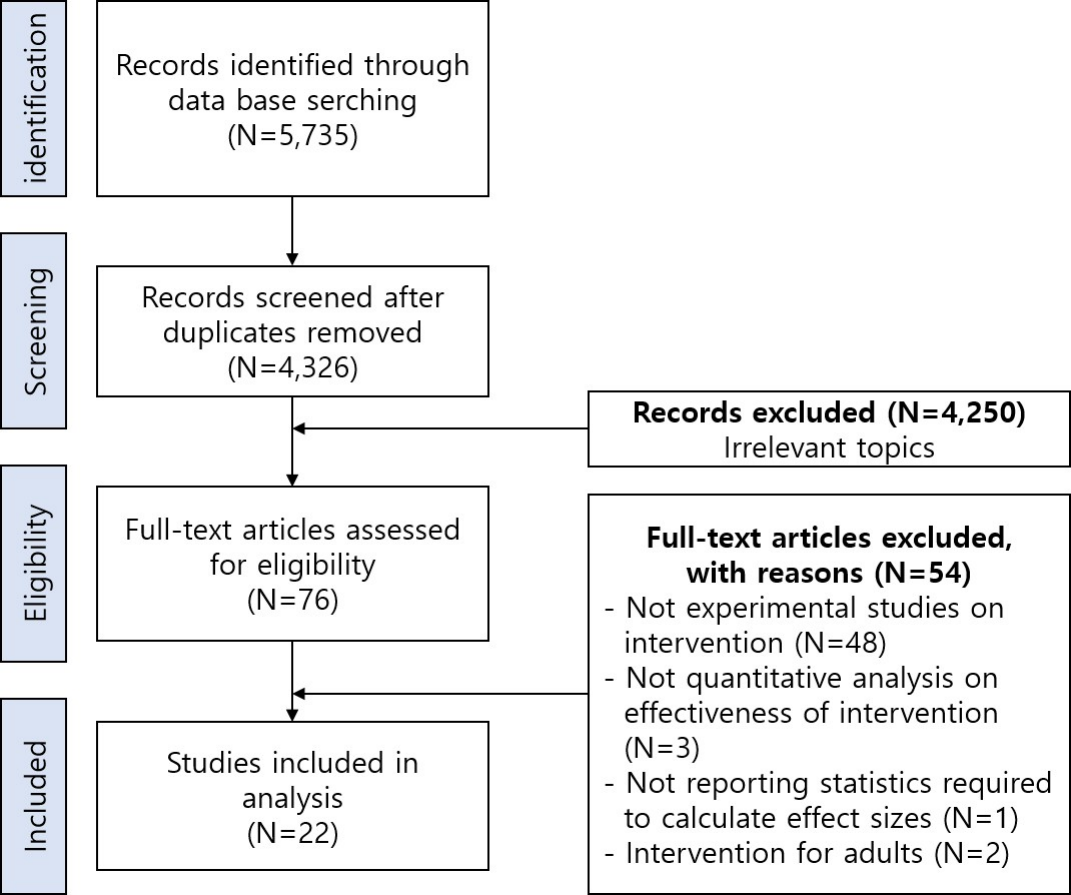
PRISMA flowchart for data collection.

We selected intervention studies on SEL programs delivered to children and adolescents before adulthood and measuring their effectiveness quantitatively by following procedures (Fig 1). First, relevant studies were identified through three Korean major research data repositories, which are the most frequently used database to conduct systematic review on Korean articles [8]: RISS (Research Information Sharing Service; https://www.riss.kr), KISS (Korean studies Information Service System; https://kiss.kstudy.com), and Nurimedia DBpia (https://www.nurimedia.co.kr). We set *social-emotional* as a term necessary to be included in the title, abstract, and keywords from each study, with combination of following nouns such as *learning*, *education*, and *competence*. While searching, we did not put a limit on the date of publication, whereas we only included peer-reviewed articles to guarantee the quality of selected literature. Through this process, the corresponding author collected literature initially 4,326 articles published by December 2020 after duplicates removed. Second, the corresponding author screened the initially collected materials at the level of titles, abstracts, and keywords. 4,250 articles with irrelevant topics were excluded, since they did not contain the phrases *social-emotional learning*, *social-emotional education*, *or social-emotional competence* in their titles, abstracts, or keywords.

Third, the corresponding author and the third author independently reviewed screened articles (N = 76) at the level of full-text to decide whether they fulfill including criteria. There were four inclusion criteria: (1) The participants of studies should be children and adolescents (PreK-12). (2) The SEL programs used in each study should emphasize the development of social-emotional competences set by CASEL [2]. (3) The studies should implement SEL and measure its effects quantitatively. (4) The studies should properly report the statistics required to calculate the effect sizes. We also suggested exclusion criteria and the number of articles excluded by each criteria was as follows: (1) The study did not conduct an experiment on the effectiveness of SEL programs aimed to enhance students’ social-emotional competences (N = 48). (2) The study did not conduct quantitative analyses on effectiveness of intervention (N = 3). (3) The study did not report statistics required to calculate effect sizes (N = 1). (4) The intervention used in the study was targeting adults (N = 2). During this process, 20% of randomly chosen articles were used for extracting the inter-observer agreement (IOA) to evaluate whether it was screened following the inclusion and exclusion criteria, and it reached full agreement between authors (100%). Based on these criteria, 22 studies were finally selected. Among them, Shin [31] presented effectiveness of two independent SEL programs in one research. We thus regarded Shin [31] as two independent studies for analyses.

### Coding procedures

#### A coding system for quality analysis

The current study implemented concrete indicators for the quality analysis based on EBI of Kratochwill & Stoiber [27]. Kratochwill & Stoiber [27] suggest a manual to review diverse prevention and intervention programs proven to be effective and utilize those programs for the next sessions. This manual has been applied to school-based suicide prevention programs [32], school dropout prevention programs [33], and family-school connection programs [34]. 18 questions were developed to investigate the quality of a statistic analysis, measurement, controlled group design, intervention fidelity, and external validity. We answered “1 (point)” or “0 (point)” to each question, “1 (point)” indicating the study has fully met each criteria whereas “0 (point)” has not. Then, we calculated the sum and proportion of studies coded “1 (point)” in each item.

For the reliability of the coding system, the second and third authors participated in the coding procedure. The second author initially completed all the items in the coding system, and specifically explained the coding method to the third author. Then, the third author reviewed the results that were firstly coded by the second author, checking for the items showed disagreements. The two coders went through the process of additional review and sufficient discussion regarding the inconsistent items, resulting in consensus at the end. Through these procedures, the reliability of coding for quality analysis was calculated as 99.6%.

#### A coding system for meta-analysis

A coding frame for the meta-analysis was developed as Table 1. We specified controlling variables into participants-, program-related variables, and types of learning outcomes, referencing Kim & Lim [9] and Seo et al. [28]. Coding for the meta-analysis went through the same as that for the quality analysis, and the reliability of coding was turned out to be 98.8%.

**Table 1:** A coding system for effect size analyses.

Study Component	Code	Details
Participants	School Level	(1)Kindergarten, (2)Elementary School, (3)Middle School, (4)High school
	Group Composition	(1)Students without disabilities only, (2)Students with disabilities only, (3)Inclusive group (integrating students with and without disabilities)
Program	Type of Program	(1)Curricular (SEL integrated with subjects), (2)Extra-curricular (SEL independent from subjects)
		(1)Program proven effective by prior studies, (2)Program developed by researcher(s)
	Number of Sessions	(1)Under 10, (2)11-20, (3)Over 21
	Session length	(1)Under 40min, (2)41-50min, (3)Over 51min
Learning outcomes		(1)Social-emotional competence in general, (2)Self-awareness, (3)Self-control, (4)Social awareness, (5)Relationship skills, (6)Responsible decision making, (7)Academic skills

#### Publication bias check

Prior to the effect size analyses of selected literature, we analyzed the publication bias, the tendency in which only studies with large effect sizes are reported and published. The sensitivity of publication bias was calculated by the trim-and-fill method. The publication bias was verified by checking whether there is no difference between funnel plot before and after the trill-and-fill conducted in funnel plot, and whether the adjusted symmetrical line did not show a statistically significant change. As a result of verifying the Egger’s test, slope of the bias was 0.23 (SE of bias = 0.62, Intercept = 0.19, *t* = 0.38, *df* = 29) and the *p*-value was 0.710. From this result, it was valid to conclude that there was no significant publication bias to synthesize the studies selected for the meta-analysis (*p* > 0.05).

#### Effect size synthesis and a random effect model

To synthesize the effect size of selected literature, we used ‘*meta*’ and ‘*metafo*r’ packages in the *R* studio program [35]. Specifically, Hedges’ *g* was calculated because this effect size is more appropriate when the sample size of each study is small [36]. We followed the Hedge’s *g* effect size calculation presented in Borenstein et al. [37]. The individual effect sizes along with 95% confidence intervals were calculated following the equations:

$$g=d\left(1-\frac{3}{4df-1}\right),\;d=\frac{\bar{D_{T}}-\bar{D_{C}}}{S_{p}},\;S_{p}=\sqrt{\frac{\left(n_{1}-1\right)S_{T}^{2}+\left(n_{2}-1\right)S_{C}^{2}}{\left(n_{1}+n_{2}-2\right)}}$$

*Note*. *n*_*c*_ = sample size of control group, *n*_*t*_ = sample size of treatment group, *S*_*p*_ = pooled standard deviation within treatment and control group, $\bar{D_{t}}=$ mean difference of pre- and post- score of treatment group, $\bar{D_{c}}=$ mean difference of pre- and post- score of control group

In each study included in the meta-analysis, there were more than one individual effect sizes because researchers usually verified the effectiveness of SEL intervention by using two or more learning outcomes. We aggregated effect sizes within each study and use all study-level effect sizes to calculate the global effect size as Cooper [35].

**Fig 2:**
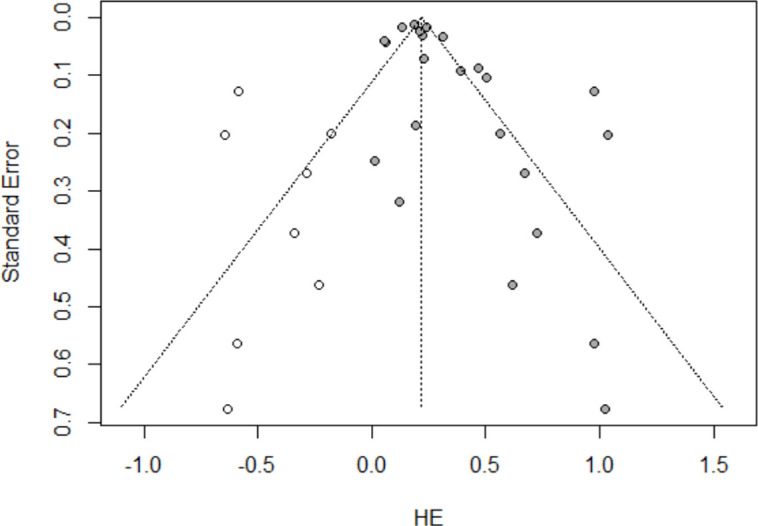
A funnel plot to confirm heterogeneity.

In the current study, a random effect size model was adopted since the heterogeneity among studies included in the meta-analysis was identified. Heterogeneity can be examined through a funnel plot (see Fig 2), a visual method to confirm heterogeneity across different studies. When the direction of the effect sizes is constant and the confidence intervals are mostly overlapping, researcher can state that the groups are homogeneous [37]. In this study, the degree of overlap of the confidence intervals of the effect sizes presented in the forest plot was identified, which corroborates heterogeneity. Additionally, the *I*-square homogeneity test was performed in order to statistically verify the heterogeneity of included studies [38]. The value of *I*-squared over 75% indicates a large heterogeneity [37]. For our results, the value of *I*-squared was turned out to be 85% (*tau-*squared = 0.053, *p* < 0.01). Because of its large heterogeneity, we decided to adopt a random model to synthesize effect sizes.

After the effect size synthesis, Q-tests were performed to confirm subgroup differences by diverse controlling variables. We additionally operated post-hoc effect size comparisons by using paired Q-tests for variables that showed significant subgroup differences in initial Q-tests to present which pairs of effect sizes were significantly different.

## Results

### Study characteristics

Table 2 shows the descriptive characteristics of study included in analyses. We reported school level, group composition, the number of participants, name and type of each program, the number of sessions, session length, and learning outcomes of each study.

**Table 2:** Descriptive of study characteristics.

Study	Lev.	Group	N	Program	Type	Se.	Len.	Dependent Variables
A	K	WD	T: 44 C: 36	Art -based SEL	C+E	10	40	In general
B	M	D	T: 15 C: 15	Strong Kids program	Ex+E	15	45	In general; self-control; social awareness
C	M	WD	T: 151	Personality education program in ethics class	C+E	9	45	Self-awareness; social awareness; relationship skills; responsible decision making
D	E	WD	T: 27	SEL	Ex+E	11	40	Self-awareness; self-control; social-awareness; relationship skills
E	E	WD	T: 52 C: 50	Emotional intelligent education using advertisement	Ex+D	12	50	Self-awareness; self-control; relationship skills; academic skills
F	E	D	T: 18 C: 18	Special education school-based SEL	Ex+D	17	-	In general; self-control; social awareness; relationship skills; responsible decision making
G	E	D	T: 5	PATHS program	Ex+E	29	40	In general; self-control; responsible decision making
H	H	WD	T: 99 C:105	SEL in English class	C+E	10	50	Academic skills
I	E	WD	T: 61 C: 58	SEL linked to academic instruction	C+E	12	40	In general; self-awareness; social awareness; relationship skills; academic skills
J			T: 57 C: 58	Strong Kids program	Ex+E			
K	E	WD	T: 18 C: 12	Agricultural experience program	Ex+E	12	60	In general; self-awareness; self-control
L	E	WD	T: 25 C: 25	Dancing program for SEL	C+D	20	60	Self-awareness; self-control; social awareness; relationship skills
M	M	I	T: 25	SEL in Korean class	C+D	21	45	Self-control; academic skills
N	E	WD	T: 10 C: 10	SEL	Ex+E	8; 16	120; 240	Self-awareness; relationship skills
O	K	WD	T: 34 C: 31	Early Childhood SEL program using story books	C+D	-	-	Self-control; social awareness; relationship skills
P	H	WD	T: 68 C: 79	SEL	Ex+E	9	50	In general; self-awareness; self-control
Q	E	WD	T: 20	Body-based awareness SEL	Ex+E	20	20	Self-awareness; self-control; social awareness; relationship skills
R	E	WD	T: 21 C: 21	Mindfulness based SEL	Ex+D	39	20	Self-awareness; self-control; social awareness; relationship skills
S	E	WD	T: 27	Integrative program of character education	Ex+E	25	-	In general; self-control
T	E	WD	T: 46 C: 46	SEL for elementary students	Ex+D	12	60	Self-awareness; self-control
U	E	I	T: 25	SEL in ethics class	C+D	19	40	In general; academic skills
V	E	WD	T: 30 C: 29	SEL in music class	C+D	12	70	In general; self-control; social awareness; responsible decision making
W	E	I	T: 80 C: 73	SEL with collaborative learning	Ex+D	16	40	Self-awareness; self-control; social awareness; relationship skills

*Note*. Lev.: School level (K: Kindergarten, E: Elementary, M: Middle, H: High school), N: number of participants (T: Treatment group, C: Control group). Group (I: Inclusive setting, WD: Students without disability, D: Students with disability), Type (C: Curricular, Ex: Extra-curricular, E: Effect proven, D: Developed by researchers), Se.: Number of sessions. Len.: Session length in minutes.

### Analysis of quality indicators

Table 3 shows the result of the first research question. To begin with, ‘Units of analysis’ confirms whether units of analysis were identical with an intervention level of each program. All included studies provided interventions in the group-level, and they also analyzed the effectiveness of the programs in the same level. ‘Family-wise error rate (FWER)’ indicates whether the study controlled the increased probability of Type 1 error when a multitude of statistic analyses were conducted. Only one among all selected studies controlled FWER by using the Bonferroni method, implying that 21 studies testing a multitude of hypothesis at once by using a limited number of assessment tools may inflate Type I errors. Furthermore, Lewis-Snyder et al. [34] suggested it is important to gather a sufficient size of sample in order to control Type II errors. Although there is no absolute standard to determine “a sufficient sample size,” Gall, Gall, and Borg [39] recommended that at least 15 individuals be included in each group of an experimental study to demonstrate the effectiveness of the program used. 20 out of 23 studies fulfilled this criterion by allotting over 15 of students for each group.

**Table 3:** A quality analysis on selected studies.

Area	Item	Number(proportion) of studies satisfied	Studies satisfying each criterion
Statistical Analysis	Units of Analysis	23(100.00%)	A~W
	FWER	1(4.35%)	E
	Sample Size	20(86.96%)	A~F, H~J, L, M, O~W
Measurement	Reliability	19(82.61%)	A, B, D~K, M, N, P~S, U~W
	Validity	4(17.39%)	D, F, G, U
	Multi-method	5(21.74%)	D, F~H, M
	Multi-rater	10(43.48%)	A, D, F~H, M~O, R, U
	Follow-up test	4(17.39%)	E, O, P, R
Control Group	Random Allocation	1(4.35%)	L
	Group Homogeneity	13(56.52%)	A, B, E, H~L, N, P, R, V, W
	Dropout Rate	16(69.57%)	A, B, E, F, H~L, N~P, R, T, V, W
Intervention Fidelity	Supervision	10(43.48%)	B, E~G, I, J, N, P, U, W
	Intervention Process	22(95.65%)	A~N, P~W
	Manual	19(82.61%)	A~D, F~H, J~N, P~S, U~W
	Recording	5(21.74%)	B, F, G, N, W
	Training	6(26.09%)	A, B, I, J, L, W
External Validity	Selecting Participants	11(47.83%)	B, E~H, K~N, P, W
	Characteristics of Participants	10(43.48%)	A, B, F, G, K, M, N, R, U, W

The results of a quality analysis on measurement use were as follows. According to the results of the ‘Reliability’ and ‘Validity’ items, 19 studies reported the reliability of each assessment tool, whereas only 4 studies reported the validity. ‘Multi-method’ refers to the use of more than one methods such as an interview, self-report, or observation in order to corroborate the effectiveness of programs, and 5 studies fulfilled this criterion. ‘Multi-rater’ indicates the participation of more than one raters in measurement, and 10 studies met this indicator. Reviewing the result of the ‘follow-up test’ item, in addition, only 4 studies conducted follow-up tests to demonstrate the effectiveness of the programs 2–8 weeks after the intervention sessions were terminated.

Focusing on the ‘Random allocation’ item from the ‘Control group’ area, control groups were designed in 16 studies, while only one study allocated students to each group at random. The ‘Group homogeneity’ indicates that there were no significant differences between treatment and control groups before implementing the program, by operating t-tests or processing covariates of pre-scores. The result showed that 13 studies conducted a statistical processing to secure the group homogeneity. ‘Dropout rate’ reveals whether there were no significant differences in dropout rates between experimental and control groups, but none of the studies had participants suspending the intervention before being completed.

For the intervention fidelity, the ‘Supervision’ item asks whether the plans for the intervention were supervised by professional educators, and 10 studies fulfilled this criterion. The ‘Intervention process’ indicates whether the study reported the number of sessions and session length, and only one study missed this information. The ‘Manual’ for the intervention reveals what instructional tools were utilized as well as what activities were implemented in each session, and 19 studies reported the concrete manual for the intervention. The ‘Recording’ item identifies whether at least one sessions during the intervention were recorded to evaluate the intervention fidelity, and 5 studies fulfilled this criterion. The ‘Training’ item decides whether the study reported the training process for interventionists, and 6 studies reported this.

Lastly, the external validity is an indicator for the detailed description of research process in order to apply it into other research settings. Among 23 studies, 11 studies properly reported the selection process of participants, and 10 studies reported the demographic characteristics of participants to check the external validity.

### A global effect size

**Fig 3:**
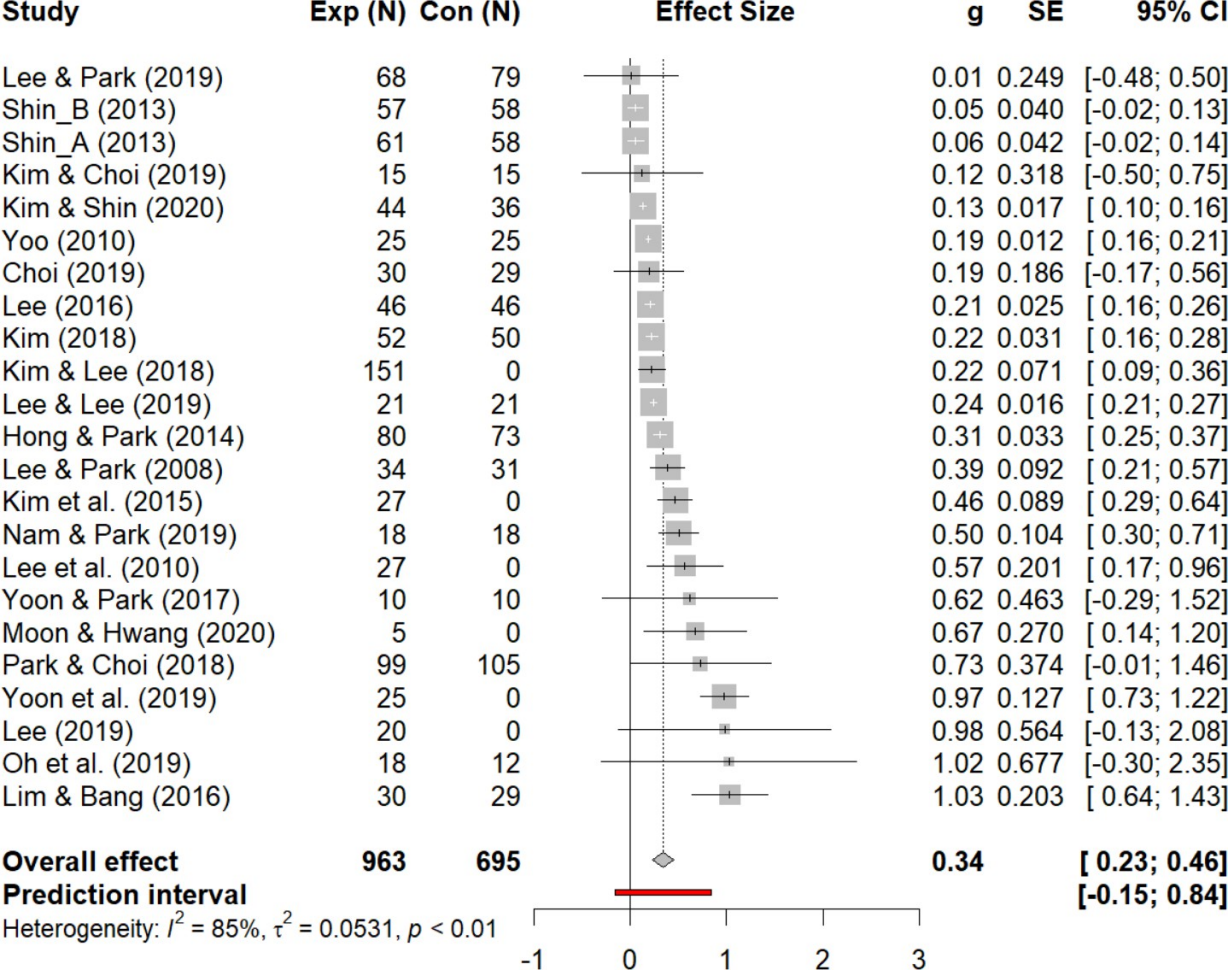
A forest plot for meta-analysis.

The results of the meta-analysis to solve the second and third research questions were as follows. The forest plot from Fig 3. shows the global effect size and the effect sizes of each article included in the analyses. The total effect size of random model was turned out to be *g* = 0.27 (*p* < .001), which indicates a medium effect (0.2 < *g* < 0.8), and its 95% confidence interval was situated between 0.23 and 0.46.

### Effect sizes by controlling variables

Table 4 presents the effect sizes by controlling variables with Q-statistics to confirm subgroup differences, and Table 5 shows the results of post-hoc effect size comparisons for the variables that showed significant subgroup differences in initial Q-tests. By the school level of participants, there were no statistically meaningful subgroup differences (*Q* = 1.50, *df* = 3, *p* = .692). By the composition of student groups, there were statistically meaningful subgroup differences (*Q* = 6.87, *df* = 2, *p* = .032). To be specific, groups integrating students both with and without disabilities had the largest effect size (*g* = .58), and groups of students with disabilities (*g* = .47) were more effective than those without disabilities (*g* = .32). From the results of post-hoc effect size comparison, there was a statistically meaningful difference only between groups of students without disabilities and groups of integrating students both with and without disabilities (*Q* = 6.50, *df* = 1, *p* = .011). By the type of SEL programs, there were no statistically meaningful subgroup differences between curricular and extra-curricular (*Q* = 2.98, *df* = 1, *p* = .084) as well as between effect proven and developed by researchers (*Q* = 0.65, *df* = 1, *p* = .421). By the number of sessions, there were statistically meaningful subgroup differences in the effectiveness of SEL programs (*Q* = 7.96, *df* = 2, *p* = .020). The results of post-hoc effect size comparison showed that programs under 10 sessions had significantly smaller effect sizes than those with 11–20 sessions (*Q* = 5.06, *df* = 1, *p* = .024) and over 21 sessions (*Q* = 7.39, *df* = 1, *p* = .007). By the session length, there were statistically meaningful subgroup differences within dependent variables (*Q* = 13.59, *df* = 2, *p* < .001). From the results of paired Q-test, there was a statistically meaningful difference between programs under 40 minutes per session and those over 51 minutes per session (*Q* = 11.46, *df* = 1, *p* < .001). Lastly, by the type of learning outcomes, there were no statistically meaningful subgroup differences (*Q* = 10.10, *df* = 6, *p* = .121).

**Table 4:** Effect size analyses by controlling variables.

		Effect size					Test for subgroup difference		
		K	*g*	SE	95%CI-L	95%CI-U	*Q*	*df*	*p*
Participant									
School Level	Kindergarten	5	0.28	0.10	0.10	0.47	1.50	3	.682
	Elementary	124	0.38	0.04	0.30	0.45			
	Middle	18	0.36	0.12	0.11	0.60			
	High	6	0.17	0.23	-0.28	0.62			
Group Composition	Without disabilities	117	0.32	0.04	0.24	0.40	6.87	2	.032
	With disabilities	19	0.47	0.13	0.22	0.72			
	Inclusion	17	0.58	0.09	0.41	0.76			
Program									
Program Type	Curricular	48	0.24	0.03	0.18	0.29	2.98	1	.084
	Extra-curricular	105	0.42	0.06	0.31	0.53			
	Effect proven	86	0.38	0.05	0.28	0.49	0.65	1	.421
	Developed by researchers	67	0.30	0.02	0.26	0.34			
Number of Sessions	Under 10	16	0.15	0.07	0.01	0.28	7.96	2	.020
	11–20	97	0.36	0.04	0.28	0.45			
	Over 21	28	0.41	0.05	0.32	0.50			
Session length	Under 40min	75	0.40	0.05	0.30	0.50	13.59	2	.001
	41-50min	31	0.30	0.06	0.19	0.42			
	Over 51min	30	0.19	0.02	0.16	0.22			
Learning Outcomes									
	Social-emotional skills in general	14	0.32	0.05	0.23	0.42	10.10	6	.121
	Self-awareness	36	0.25	0.11	0.03	0.47			
	Self-control	36	0.29	0.05	0.20	0.38			
	Social awareness	21	0.58	0.22	0.15	1.01			
	Relationship skills	30	0.20	0.04	0.12	0.29			
	Responsible decision making	5	0.31	0.08	0.14	0.47			
	Academic skills	11	0.32	0.05	0.23	0.41			

Note. K: the number of effect sizes, *g*: Hedges’ *g*, SE: Standard Error, CI-L: Confidence intervals lower limit, CI-U: Confidence intervals upper limit.

**Table 5:** Post-hoc effect size comparisons.

Variable	Paired comparison	Test for subgroup difference		
		*Q*	*df*	*p*
Group Composition	Without disabilities vs. With disabilities	1.03	1	0.310
	Without disabilities vs. Inclusion	6.50	1	0.011
	With disabilities vs. Inclusion	0.49	1	0.484
Number of Sessions	Under 10 vs. 11–20	5.06	1	0.024
	Under 10 vs. Over 21	7.39	1	0.007
	11–20 vs. Over 21	0.59	1	0.443
Session length	Under 40min vs. 41-50min	1.10	1	0.293
	Under 40min vs. Over 51min	11.46	1	< .001
	41-50min vs. Over 51min	2.35	1	0.126

## Discussion

### Quality of experimental studies on SEL

The main findings in regard to the quality analysis and directions for future research and practice were discussed below. First, the intervention studies on SEL should consider FWER to accurately indicate the effectiveness of the instructions used. Almost all studies included in the analysis (N = 21) tested a multitude of hypothesis at once by using a limited number of assessment tools, which may inflate Type I errors. Only Kim [40] controlled FWER by using the Bonferroni method, the most frequently used control method. FWER are dramatically increased as the number of statistic analyses increases [28]. Experimental studies on SEL tend to have more than one dependent variables since SEL often aims to improve diverse social-emotional and academic competences of students at once. Therefore, the study which demonstrates the effectiveness of a program on various educational outcomes with a limited number of assessment tools should conduct additional statistical procedures to control the inflated Type I error.

Second, for the quality analysis on measurement use, it was found that only 17% of studies reported the validity of assessment tools. Reliability and validity are two main indicators for deciding the interior criteria of assessment tools [28]. If a researcher utilized an assessment tool outside of its original purpose, readers would not be able to trust the measures taken by it. It is thus imperative to use assessment tools that guarantee high validity and report this information on the study.

About 79% of included literature did not use multiple methods other than a survey to measure learning outcomes of participants. As survey results are subject to changes from different raters, the results from other methods such as observation and interviews can support survey results. If observations or interviews were not available, measurement by multi-raters would be a recommended alternative. According to Blewitt et al. [11], there was a significant difference between effectiveness of SEL reported by parents and that by teachers. Based on the prior study, it is possible to insist that multi-raters should be incorporated in the measurement process in order to report unbiased results.

In addition, only 4 studies conducted follow-up tests which were taken after the intervention was terminated. SEL pursues a better preparation for careers and adulthood, as well as immediate improvement of social-emotional competences during school lives [41]. Follow-up tests are thus required to measure how SEL affects students in the long term. In Taylor et al. [4], for instance, it was proved that SELs for kindergarten through high school aged students had great positive effects on overall well-being in adulthood. Likewise, Korean or non-Western intervention studies should also aim to corroborate the long-term effects of SEL on participants in order to fully realize the purpose of SEL.

Third, about 70% of studies included control groups within the research design. The effectiveness of the program can be confused with natural maturation or a simple learning effect when the control group is missing [28]. Thus, it is important to divide participants into treatment and control groups to accurately assess the effectiveness of the program. However, randomly allotting the participants into a control group may lead to ethical problems, in that students included in the control group would not have any benefits even though they participated in the research. To solve this problem, we suggest that the control group be converted into a comparative group in which students are provided with an alternative intervention. In other words, the treatment group can be designed for measuring the effectiveness of a newly developed program, whereas the comparative group is provided with a program proven effective in prior studies. By using this method, researchers can not only establish a controlled experiment but also fulfill the educational needs of all participants.

Fourth, the quality analysis in intervention fidelity showed that under 30% of the included articles recorded sessions during interventions. Intervention fidelity is important to confirm whether the intervention was operated in accordance with its plan, and professionals need to visit the intervention sessions or watch the recordings of several sessions to accurately evaluate their fidelity. In particular, in countries where SEL programs have not been actively implemented and where the EBP of SEL is not well-established, the importance of evaluating intervention fidelity cannot be overemphasized during the implementation of SEL. Therefore, experimental studies should properly report what sources were provided for the professionals and whether a part of the intervention sessions were recorded to confirm the intervention fidelity.

Furthermore, only 6 studies reported how the interventionists were trained, even though the professionality of interventionists mainly decides the overall quality of the intervention. According to Farmer et al. [42], teachers should acquire strategies to improve students’ engagement, substitute problem behaviors with positive ones, and deal with social dynamics in classrooms so as to maximize the adjustment in social, emotional, and academic domains. To acquire and be proficient with those strategies, a long-term training is definitely needed. If teachers did not fully experience the training process, readers cannot guarantee the fidelity of interventions. We thus suggest that studies report the training process of interventionists to assure that they are qualified to implement SEL programs.

Fifth, the quality analysis on external validity showed that under 50% of the selected studies properly described the selection process and/or demographic characteristics of participants. External validity indicates the exhaustive description of research processes in order to apply identical programs to other settings, and the selection process as well as characteristics of participants are integral information to confirm it [43]. The external validity is necessarily required to decide the EBP which aims to apply the results of scientific studies into diverse educational settings. The following empirical studies on SEL thus should exhaustively describe the information of participants to secure higher external validity.

### Effectiveness of SEL

The main findings regarding the meta-analysis and directions for future research and practice were discussed as follows. To begin with, the global effect size of included articles is 0.27, which is situated in a middle-sized effect but close to a small-sized effect (*g* < 0.2). This figure is similar to a global effect size (*g* = 0.30) calculated by Durlak et al. [10]. However, the global effect size Durlak et al. [10] calculated included the effects of SEL not only on social-emotional competencies and academic achievement but also on levels of self-esteem, emotional and behavioral disorders, and prosocial behaviors, which are not incorporated in the current study. In this sense, effect size analyses by diverse controlling variables, including learning outcomes, are highly required to avoid misinterpretation of results.

The results of effect sizes by participant-related variables showed that SEL had larger effects on students in the order of elementary schools, middle schools, kindergartens, and high schools, but they had no statistical differences. According to Murano, Sawyer, & Lipnevich [12], the effect sizes of SEL for kindergarten children were situated between *g* = 0.32~0.50, which is somewhat larger than those of the current study (*g* = 0.28). Murano et al. [12] suggested that SELs for early children be planned with a connection to their home, as they spend much time there. However, the SEL programs for kindergarteners included in the current meta-analysis focused on the connection with content knowledge children acquired as opposed to on the connection with homes and parents. Therefore, the following studies on SEL programs for the early children should develop activities to connect the kindergarten and students’ families.

In addition, as the number of effect sizes in high school SEL programs was small (N = 6), their effects turned out to be statistically insignificant. High school education usually concentrates on academic development and entrance into higher education, not on students’ social-emotional development, which often leads to high school SEL being ignored [44]. However, adolescents, due to their developed cognitive abilities, are able to acquire and master social-emotional skills that are not expected of elementary students [45]. For instance, Durlak et al. [10] showed that SEL for adolescents reduced their behavioral problems, and improved social adjustment, school engagement, and academic achievement. Hence, the SEL targeting high school students should be more actively implemented in diverse educational settings.

By the group composition, effect sizes of inclusive groups were significantly larger than groups comprised only of students without disabilities. The SEL aims to educate students to solve problems in social settings with great responsibility by understanding other people as well as themselves. To achieve this goal, experiences with students from diverse backgrounds is recommended, which can explain the large effect size of students in inclusive groups. However, SEL including students with diverse needs should be planned with caution, considering the three following factors [46]: (1) Although students with learning disabilities, emotional and behavioral disorders, and intellectual disabilities have different diagnoses, they may have common academic, social, and psychological needs. It is important not to be obsessed with labels of disabilities while planning SEL programs. (2) In contrast, students with the same type of disability may report diverse problems. Teachers thus implement instructions which fit with the special educational needs of each student. (3) There are lots of students who are not yet diagnosed with a disability, but nonetheless have difficulties in adjustment. Teachers need to identify students at risk for disabilities, and design SEL programs to support them. As these factors may be demanding to interventionists, it is important for them to improve professions on educational services for students with disabilities before implementing the intervention.

The results of effect sizes by program-related variables showed that types of programs did not significantly impact the effectiveness of each program. Specifically, comparing the effectiveness between programs incorporated into and those operated separately from general curriculum, the number of SELs operated within general curriculum was marginal compared to the others. However, it is now recommended that SELs integrated with subject education be more actively implemented in school settings for the following two reasons. First, SEL aims to help students in “overall adjustment,” including academic adjustment as well as socioemotional adjustment [2]. That is, SEL programs that integrate social-emotional skills with academic skills better meet with its original purpose. Second, SEL programs are usually implemented not for selective interventions but for general education targeting all students in school settings [5, 11, 46]. In order to improve efficiency of school curriculum operation, SEL programs should be operated in accordance with other subjects in general education. Therefore, future research should work more on developing effective SEL programs with diverse subjects as a part of general and inclusive education.

The effect sizes of SEL programs over 10 sessions were significantly larger than those comprised of less than 10 sessions. This suggests that students should participate in the SEL program for an extended period of time (i.e., at least 10 sessions) in order to maximize its effectiveness. Furthermore, SEL programs with lessons 40 minutes or less per session showed significantly larger effect sizes than those with lessons over 50 minutes. A Lesson of 40 minutes or less reflects the typical time duration of one lesson in elementary schools. As most participants included in this study were elementary school students, this result may reflect the majority of participants. If the highest proportion of participants were not comprised of elementary school students, the findings about session length might be different from the current result. Thus, we can tentatively conclude that the most effective instructions should be planned in the unit of time which students are most accustomed to and which allow them to keep concentrating on the tasks.

The results of effect sizes by learning outcomes showed that the effect of SEL programs on social awareness was the largest whereas that on relationship skills was the smallest, although the difference in effect sizes among learning outcomes was not statistically meaningful. Discussing a relatively low effect size in relationship skills (*g* = 0.20), the abilities to communicate, cooperate with, and support others [2], it was similar with effect sizes of prosocial skills and behaviors calculated by other meta-analytic studies (*g* = 0.13 [4], 0.24 [10], and 0.33 [5]). In all of those studies, the effect of SEL programs on relationship skills was smaller than the other social-emotional skills such as self-awareness, self-control, and social awareness. This might be mainly because skills to communicate and cooperate with others require students to understand the social dynamics among social groups, which can be difficult and complex to master within a short period of time [47]. However, it is also necessary to find a better practice to improve relationship skills in SEL programs. For instance, the ‘Caring School Community (CSC)’ program has been proven effective to enhance relationship skills, altruistic behaviors and prosocial behaviors by engaging in activities such as setting classroom rules, implementing collaborative instructions with higher grade students, and communicating with parents about students’ school lives [48]. In-service teachers can utilize some of these activities within their SEL programs to successfully manage classroom social dynamics and ultimately increase the relationship skills of students.

Lastly, the effect of SELs on academic achievement was turned out to be satisfactory (g = 0.32), while the number of its effect sizes was somewhat marginal (N = 11). The effect sizes on academic performance calculated in prior meta-analyses (*g* = 0.18 [11], 0.33 [4], 0.27 [10], 0.19~0.25 [13], and 0.28 [5]) were also similar to the current study. However, the measures of academic achievement in prior studies [4, 10, 11, 13] incorporate the actual academic performance in school curriculum such as math, reading, and science, whereas those measured in the current study were mostly on academic engagement and motivation. Although academic engagement and actual performances are highly correlated [3], it is still required to demonstrate that SEL directly contributes to improving actual academic performances through future studies.

### Limitations

The limitations of the current study were as follows. To begin with, as the number of studies included in analyses was insignificant (23 SEL programs), the interpretation of the results needs a meticulous care. Second, while the current study revealed the effect sizes by various controlling variables to identify the EBP of SEL, we could not figure out certain instructional strategies that are effective to enhance social-emotional skills in SEL. The future research thus need to analyze the instructional methods or strategies that are often used in SEL programs, and reveal what strategies are the most effective. Third, although we categorized the types of learning outcomes based on the five important social-emotional competences that CASEL (2015) emphasized, this is neither an absolute nor the most appropriate way to classify learning outcomes of SEL programs. The effect sizes by the types of learning outcomes can be varied according to how they are categorized. Fourth, we did not consider correlations among effect sizes in the same study when analyzing them. Therefore, the future research should focus more on possible dependency among different controlling variables to fully manifest EBP of SEL programs.

## Supporting information

S1 Checklist PRISMA 2009 checklist. (DOCX)

S1 File References included in a meta-analysis. (DOCX)

S2 File A questionnaire for a quality analysis. (DOCX)
