# Supplementary material for: The quality and effectiveness of Social-Emotional Learning (SEL) intervention studies in Korea: A meta-analysis
Source: PLoS One. 2022 Jun 24;17(6):e0269996. doi: 10.1371/journal.pone.0269996 (PMC9231796; doi:10.1371/journal.pone.0269996)
Supplement: S1 File — (DOCX) [file pone.0269996.s002.docx]

**References included in a meta-analysis**

A. Kim, G. H., & Shin, T. S. (2020). Study on the development and effects of arts based social-emotional learning program on children. *The Journal of Korea Open Association for Early Childhood Education, 25*(5), 293-319.

B. Kim, M., & Choi, H. (2019). The effects of Strong Kids Program on social-emotional symptoms and functions of students with intellectual disability of special classes in middle schools. *Journal of Special Education, 26*(1), 41-68.

C. Kim, Y. G., & Lee, I. T. (2018). The effect of moral education program based on SEL. Journal of Moral & Ethics Education, 61, 53-81.

D. Kim, E. J., Jin, C. H., & Lee, S. (2015). The exploration of the effects of social and emotional learning program on the social and emotional competencies of elementary school students and their community consciousness. The Korean Journal of Educational Methodology Studies, 27(4), 511-534.

E. Kim, J. E. (2018). A study of development and effects of the emotional intelligence education program using advertising. Journal of Media Economics & Culture, 16(1), 7-56.

F. Nam, H., & Park, S. H. (2019). Effects of social emotional learning implemented in a special school on the social emotional knowledge, social emotional skills and peer interactions of students with intellectual disabilities. Korean Journal of Special Education, 54(2), 95-127.

G. Moon, S., & Hwang, S. (2020). The effects of the PATHS program on the emotional awareness and social competence of elementary students with moderate intellectual disabilities. Journal of Special Education, 27(1), 275-312.

H. Park, Y. S., & Choe, M. H. (2018). Applying social-emotional learning to teaching English in a vocational high school. Secondary English Education, 11(2), 53-78.

I/J. Shin, H. S. (2013). Classroom-based social and emotional learning linked to academic instruction: Its effects on social-emotional competences and school-related outcomes. The Korean Journal of School Psychology, 10(1), 83-110.

K. Oh, Y. A., Kim, D. S., & Park, S. A. (2019). Analysis of the emotional effects of agricultural experience program based on social emotional learning theory in elementary school students. Journal of the Korean Society of Rural Planning. 25(4), 87-97.

L. You, Y. R. (2010). The effects of teaching-learning strategies for elementary school students’ social and emotional learning in dance classes. The Korean Journal of Arts Education, 8(3), 179-194.

M. Yun, J. H., Bang, M. Y., & Kim, O. J. (2019). The effects of a social and emotional learning program integrated with Korean language education on the class participation of a middle school student at risk for emotional and behavior disorders and the emotional intelligence of non-disabled students. Journal of Emotional & Behavioral Disorders, 35(2), 177-196.

N. Yun, H., & Park, J. (2017). The effects of a social and emotional learning program on the self-concept, social behavior and communication with parents of elementary students who have siblings with disabilities. Studies on Korean Youth, 28(2), 5-33.

O. Lee, K. Y., Park, A. C. (2008). A study on the development of children’s social and emotional education program using picture books. The Journal of Child Education, 17(2), 213-222.

P. Lee, S., & Park, J. (2019). The effects of social and emotional learning program on mental health of girls in general high school. Korean Journal of Counseling. 20(5), 311-332.

Q. Lee, S. H. (2019). A preliminary study for the development of the body-based awareness social-emotional learning program. Journal of Brain Education, 23, 7-27.

R. Lee, S., & Lee, S. (2019). The effect of mindfulness based social emotional learning program on the improvement of elementary school students’ social emotional competence. The Journal of Humanities and Social Science, 10(5), 379-394.

S. Lee, I. J., Son, K., Chi, C. H., & Han, S. G. (2010). An analysis on effect of the integrative program of character education for primary school students social and emotional competencies. Journal of Moral & Ethics Education, 31, 49-82.

T. Lee, I. T. (2016). Design of emotion education program for elementary school students’ moral emotional competence. Journal of Moral & Ethics Education, 52, 63-93.

U. Lim, J. H., & Bang, M. Y. (2016). The effects of a social and emotional learning program integrated with moral education on the class participation of an elementary school student at risk for emotional and behavior disorders and the emotional intelligence of nondisabled students. Journal of Emotional & Behavioral Disorders, 32(1), 191-211.

V. Choi, M. Y. (2019). The effects of music subject related SEL program for enhancing elementary school students’ social-emotional competence. Research in Music Pedagogy, 20(1), 153-178.

W. Hong, J. A., & Park, S. (2014). Effects of a class-wide social emotional learning program on elementary students’ emotional intelligence and peer supports in inclusive classrooms. Korean Journal of Special Education, 49(2), 213-239.
