# Supplementary material for: The quality and effectiveness of Social-Emotional Learning (SEL) intervention studies in Korea: A meta-analysis
Source: PLoS One. 2022 Jun 24;17(6):e0269996. doi: 10.1371/journal.pone.0269996 (PMC9231796; doi:10.1371/journal.pone.0269996)
Supplement: S2 File — (DOCX) [file pone.0269996.s003.docx]

**A Questionnaire for a Quality Analysis**

|  | Area | | Questions | |
| --- | --- | --- | --- | --- |
| 1 | Statistical Analysis | Units of Analysis | | Were the units of analysis identical with an intervention level of each program? |
| 2 |  | FWER | | Was the family-wise error rate controlled? |
| 3 |  | Sample Size | | Did each group consist of more than 15 students? |
| 4 | Measurement | Reliability | | Was the reliability of each assessment tool reported? |
| 5 |  | Validity | | Was the validity of each assessment tool reported? |
| 6 |  | Multi-method | | Was the effectiveness of program evaluated by more than one methods? |
| 7 |  | Multi-rater | | Did more than one raters participate in the measurement process? |
| 8 |  | Follow-up test | | Was the follow-up test conducted? |
| 9 | Control Group | Random Allocation | | Were participants randomly allotted in experimental or control groups? |
| 10 |  | Group Homogeneity | | Was there a homogeneity between experimental and control groups? |
| 11 |  | Dropout Rates | | Were the dropout rates of experiment and control groups identical? |
| 12 | Intervention Fidelity | Supervision | | Did professionals supervise the plans for the intervention? |
| 13 |  | Intervention Process | | Did the study report the intervention process? |
| 14 |  | Manual | | Was there an explicit manual for the intervention? |
| 15 |  | Recording | | Did researchers record any part of the intervention? |
| 16 |  | Training | | Did the study report the training process for interventionists? |
| 17 | External Validity | Selecting Participants | | Did the study report the selection process of participants? |
| 18 |  | Characteristics of Participants | | Did the study report the demographic characteristics of participants? |
